# Feline T-Cell Receptor *γ* V- and J-Region Sequences Retrieved from the Trace Archive and from Transcriptome Analysis of Cats

**DOI:** 10.4061/2010/953272

**Published:** 2010-06-16

**Authors:** Alexander Thomas Andreas Weiss, Werner Hecht, Manfred Reinacher

**Affiliations:** ^1^Institute of Veterinary Pathology, University of Giessen, Frankfurter Strasse 96, 35392 Giessen, Germany; ^2^Department of Veterinary Pathology, Freie Universitaet Berlin, Robert-von-Ostertag-Strasse 15, 14163 Berlin, Germany

## Abstract

The variable domains of antigen receptors are very diverse and assembled in a modular system from a number of V-, D-, and J-region genes. Here we describe additional variants of V- and J-region genes of the feline T-cell receptor *γ* (TRG) as well as the corresponding RSSs retrieved from Trace Archive of feline genomic sequences. Additionally, an unusually recombined TRGV-domain containing a partial inverted repeat of the included J-region and a short interspersed element of the CAN-SINE family located within the feline T-cell receptor *γ* locus are also described.

## 1. Introduction

In the course of lymphocyte development the V-domains of T-cell receptor (TR) genes, as well as immunoglobulin genes, are somatically rearranged using two or three different regions in a process called V(D)J recombination. First a D-region, if present, is joined to a J-region then a V-region is joined to the DJ-region. Fusion with the C-region happens during RNA maturation by splicing [1, 2]. Immunoglobulin light chains, the TRG, and the TRA lack a D-region and the V-region is joined directly to the J-region. Diversity is further enhanced by imprecise joining during this process [1]. 

V(D)J-recombination is initiated by the products of the recombination activating genes 1 and 2 ( RAG1 and RAG2). They bind to the recombination signal sequence (RSS) and induce a DNA double strand break [3, 4]. The signal ends and the coding ends are then processed by the ubiquitous mechanism of non-homologous end joining (NHEJ) [5]. 

The RSSs are made up of a highly conserved heptamer, a conserved adenine rich nonamer, and a less conserved spacer of 12 ± 1 or 23 ± 1 bp. The length of the spacer is used to characterize the RSS and they are annotated 12RSS and 23RSS. During rearrangement, a 12RSS is always combined with a 23RSS. This fact is known as the 12/23-rule [1, 5, 6]. In the TRG locus, V-regions have a 3′-23RSS and J-regions a 5′-12RSS [7].

In the feline TRG repertoire four different V-region genes (fTRGV1 – 4), eight different J-region genes (fTRGJ1.1 to -1.5, fTRGJ2.1 and -2.2, fTRGJ3), and six different C-region genes (fTRGC1 to -6) have already been described [8, 9]. Compared to the known human TRG repertoire fewer V-region genes are known in the cat (4) than in humans (12– 15) [7, 10]. In contrast, cats have greater J- and C-region diversity (8 and 6 versus 5 and 2, res.) [7, 10]. 

An interesting feature of TR loci of humans and mice is that they contain Long Interspersed Elements (LINEs) and Short Interspersed Elements (SINEs) at a density below the average of the genome [11]. SINEs are short mobile DNA elements of eukaryotes. They are retrotransposons that proliferate by transcription into RNA, reverse transcription into DNA, and reintegration into the genome. SINEs are 80 to 400 bp long and need enzymes encoded by LINEs for proliferation. SINEs excluding Alu-sequences of primates are derived from tRNA and contain a promoter for DNA-dependent RNA-polymerase III. They are flanked by direct repeats. LINEs and SINEs make up more than 30% of the human genome [12, 13]. Typical SINES of carnivorous species are called CAN-SINES because they were initially identified in *Canidae* [14].

Here we describe four additional variants of V-region genes and one additional variant of J-region genes retrieved from the Trace Archive of feline genomic sequences (NCBI, Bethesda, USA). Additionally a unique construct containing a previously unknown J-region sequence and a CAN-SINE located within the feline TRG locus are also described.

## 2. Material and Methods

### 2.1. Sequence Analysis

Previously generated J-region and V-region sequences [9] were used to search the Trace Archive of feline genomic sequences (NCBI, Bethesda, USA; http://www.ncbi.nlm.nih.gov/Traces/) employing the BLAST Search algorithm (NCBI, Bethesda, USA; http://www.ncbi.nlm.nih.gov/blast/). Sequence analyses were carried out using ClustalW (EMBL-EBI, Heidelberg, Germany; http://www.ebi.ac.uk/Tools/clustalw/) and V-Quest software (IMGT, Montpellier, France; http://imgt.cines.fr/IMGT_vquest/share/textes/ [16]). GeneDoc 2.6.003 software was used for displaying the multiple sequence alignments.

### 2.2. SMART RACE for Feline TRG Sequences

We extracted total RNA from the thymus of an 8-week-old male Domestic short hair cat (died from blunt trauma) and the spleen of an 18-years-old female domestic shorthair cat (euthanized because of mammary carcinoma) with the Purescript RNA Isolation Kit (Biozym, Oldendorf, Germany) as recommended. 5′RACE was performed using the SMART RACE cDNA Amplification Kit (BD Biosciences, Heidelberg, Germany) as recommended by the manufacturer. The amplification was carried out as nested PCR using Phusion High-Fidelity DNA Polymerase (BioCat, Heidelberg, Germany) as recommended. We used primer eFTGr1 (5′- ATT GAA GGA AAC AGA ATC TCT TG-3′, position 300–322) for cDNA synthesis and primers eFTGr2 (5′- CAT TTG TGT TCT TTG CCC ATT GAC TC-3′, position 237–262) and eFTGr3(5′ GTC AGC CAG GTG TAT TTC ATG TAT GTG -3′, position 200 – 226) for consecutive rounds of nested PCR. Initial denaturation was carried out for 30 seconds at 98°C, followed by melting for 10 seconds at 98°C, primer annealing for 30 seconds and elongation for 30 seconds at 72°C, and a final elongation for 5 minutes at 72°C. Specific annealing conditions in the first round of PCR were 70°C with a touch-down of 0.5°C over 15 cycles followed by 62.5°C for an additional 21 cycles. In the second round we used 70°C with a touch-down of 0.5°C over 12 cycles followed by 64°C for 24 cycles. DNA was handled, cloned, and sequenced as described [9]. Sequences containing a CAN-SINE and one particular sequence containing a new fTRGJ-variant were submitted to EMBL Nucleotide Sequence Database: AM502837, AM502838, AM502846, and AM747390 (fTGII.2).

## 3. Results

**Figure 1:**
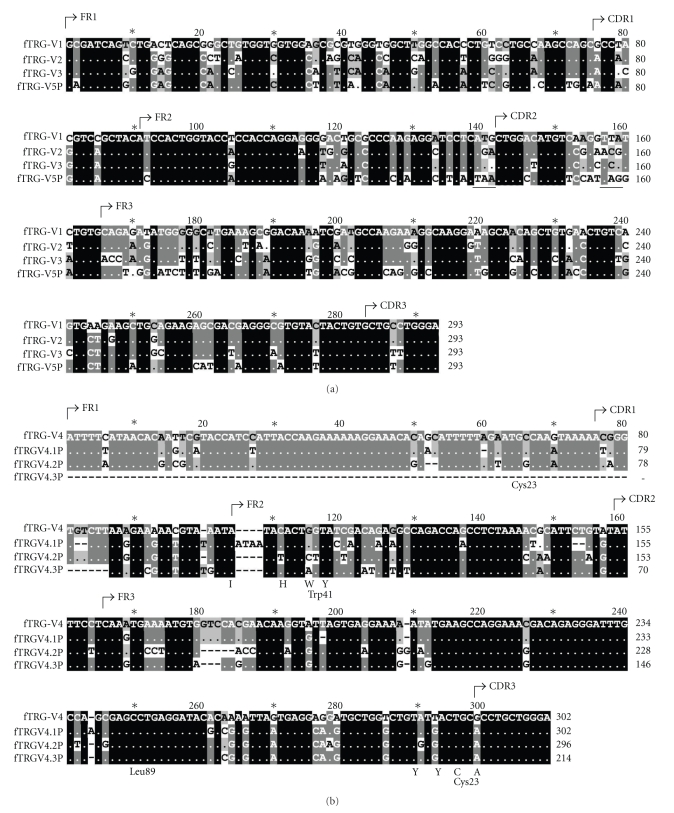
(a) Alignment of fTRGV5P (gnl|ti|663092402) with previously published V-region genes of the feline TRG. The two stop codons are underlined (bp139–141 and bp157–159). (b) Alignment of fTRGV4 and the newly identified pseudogenes fTRGV4.1P ($\underline{\text{gnl}|\text{ti}|838072588}$, $\underline{\text{gnl}|\text{ti}|827924706}$), -4.2P ($\underline{\text{gnl}|\text{ti}|643947952}$, $\underline{\text{gnl}|\text{ti}|646819523}$, $\underline{\text{gnl}|\text{ti}|644534307}$), and -4.3P ($\underline{\text{gnl}|\text{ti}|631627457}$, $\underline{\text{gnl}|ti|630610594}$, $\underline{\text{gnl}|ti|2159892447}$). Conserved amino acids together with the unique IMGT-numbering [15] and characteristic motifs are indicated below the DNA sequence.

**Figure 2:**
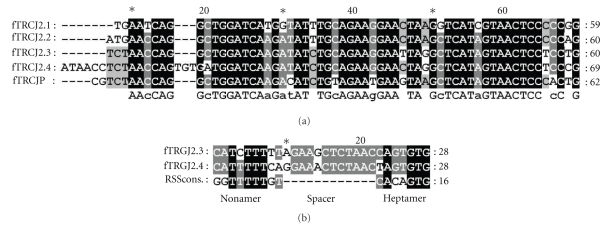
(a) Alignment of feline fTRGJ2-variants. In fTRGJ2.4 a three-base pair long insertion is present near the 3′ end. The consensus sequence is given below the original sequences. (b) Alignment of 12RSSs belonging to fTRGJ2 variants and the consensus sequence (RSScons.) [6]. In fTRGJ2.4 the potential heptamer of the RSS created by an insertion of “TGT” (5′-CAG**TGT**G-3′) is more similar to the consensus sequence than the original one. The mutation of the first residue (C→T) results in an alteration of the essential “CAC” sequence [17].

**Figure 3:**
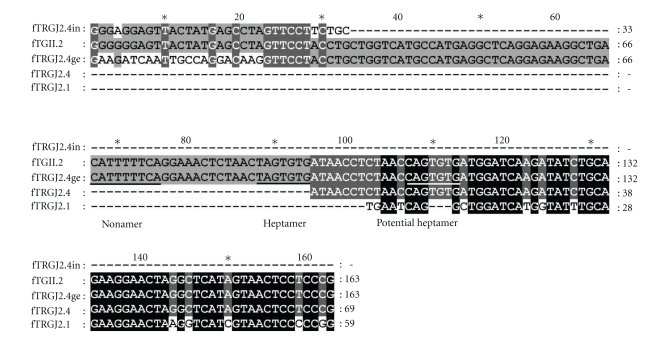
Alignment of fTGII.2 with the fTRGJ2.4, its genomic sequence (fTRGJ2.4ge: $\underline{\text{gnl}|\text{ti}|915239161}$), and fTRGJ2.4 in inverted orientation (fTRGJ2.4in). The six base pairs at the 3′ end of the inverted portion of fTRGJ2.4, are homologous in the inversion, in fTRGJ2.4 and in the genomic sequence upstream of fTRGJ2.4.

A BLAST search of the Trace Archive yielded four previously unidentified V-region genes designated as fTRGV5P ($\underline{\text{gnl}|\text{ti}|663092402}$), -4.1P ($\underline{\text{gnl}|\text{ti}|838072588}$, $\underline{\text{gnl}|\text{ti}|827924706)}$, -4.2P ($\underline{\text{gnl}|\text{ti}|643947952}$, $\underline{\text{gnl}|\text{ti}|646819523}$, $\underline{\text{gnl}|\text{ti}|644534307}$), and -4.3P ($\underline{\text{gnl}|\text{ti}|631627457}$, $\underline{\text{gnl}|\text{ti}|630610594}$, $\underline{\text{gnl}|\text{ti}|2159892447}$) (see Figures 1(a) and 1(b) and one potential J-region gene designated as fTRGJ2.3 (see Figure 2(a) besides those already described [8, 9]. One further J-region gene (fTRGJ2.4, see Figures 2(a) and 3) was identified in one clone (fTGII.2) amplified by the SMART RACE procedure described above.

Newly identified V-region genes have mutations within the coding sequence or the RSS. Upon analysis using V-QUEST, a specialised sequence alignment software for Ig, TR, and HLA, fTRGV5P had two stop codons in the correct reading frame (see Figure 1(a). Within the putative RSS an insertion consisting mostly of cytosine was found. Nucleotide homology of fTRGV5P to previously described V-region genes of the first subgroup of the fTRG [8, 9] ranged from 68 to 72 %, and to fTRGV4 it was 6 % (see Table 1). 

fTRGV4.1P, fTRGV4.2P, and fTRGV4.3P are close relatives of already described fTRGV4 [9]. Nucleotide homology of the new variants to fTRGV4 is 88, 84 and 86%, respectively (see also Table 1). fTRGV4.1P, -4.2P and 4.3P feature insertions and/or deletions that result in frame shifts and thereby alterations of amino acid residues and motifs that are vital for receptor configuration (see Figure 1(b). Those amino acid residues are the conserved cysteine23, tryptophan41, leucine89, and cysteine104 as well as the characteristic motifs “IHWY” at the beginning of FR2 and “YYC” at the end of FR3 [15]. Additionally the nonamers of the 23RSSs are substituted by cytosine- and thymidine-rich insertions. In fTRGV4.3P the first 86 bp are replaced by CT- and A-rich repeats. 

A BLAST search of the Trace Archive for fTRGJ2.2 [9] revealed a further potential J-region gene with 86 % nucleotide homology, that we designated fTRGJ2.3 (see Figure 2(a) and Table 2) ($\underline{\text{gnl}|\text{ti}|915242736}$, $\underline{\text{gnl}|\text{ti}|716919583}$, $\underline{\text{gnl}|\text{ti}|716917754}$ , $\underline{\text{gnl}|\text{ti}|836113710}$, $\underline{\text{gnl}|\text{ti}|646818114}$, $\underline{\text{gnl}|\text{ti}|964951196}$). 

When applying the SMART RACE procedure to RNA extracted from the thymus of an 8-week-old kitten we obtained a very noticeable construct (see Figure 3). This clone, fTGII.2, included a 25 bp long inverted repeat of the 3′ half of the new J-region variant fTRGJ2.4 (see also Figure 2(a)). This J-region variant is also represented in Trace Archive ($\underline{\text{gnl}|\text{ti}|915239161}$, $\underline{\text{gnl}|\text{ti}|652438676}$). The following 66 bp in 3′ direction are identical to the genomic DNA upstream of fTRGJ2.4. Consecutively in 3' direction fTRGJ2.4 follows itself. The 30 bp upstream of the inverted repeat to the 3′ end of the V-region are nither identical to the genomic sequence downstream of fTRGJ2.4 nor to fTRGV1. Therefore, they could be regarded as N-region. However, no potential 12RSS could be found in the genomic sequence downstream of fTRGJ2.4 that could have served as target for RAG in the initiation of recombination.

**Figure 4:**
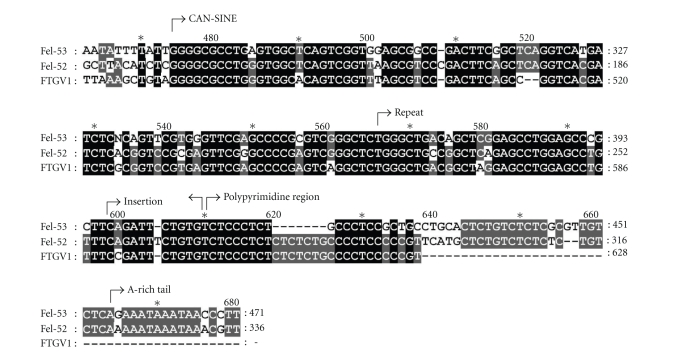
Alignment of the CAN-SINE amplified by a SMART RACE procedure with typical CAN-SINES of cats. The clone FTGV1 is depicted as a complementary strand in relation to the TRG genes. The CAN-SINE has an insertion prior to the polypyrimidine region and a direct repeat after the tRNA-related part that are typical in feline CAN-SINEs [14].

Additionally we cloned and sequenced an unspliced pre-RNA containing the constant region variant fTRGC4 and the genomic sequence in 5′ direction. At the 5′ end of these sequences a short interspersed element with features typically found in *Canidae* and carnivores in general (CAN-SINE) could be identified (see Figure 4). It is positioned 468 bp upstream of the beginning of the C-region gene and is oriented in the opposite direction. This SINE is 164 bp long and has a fragmented polypyrimidine region. When comparing it to other SINEs known in the cat genome, homology is highest to Fel-52 (90%, AW646738) and Fel-53 (85%, AW646884) followed by Fel-1 (81%, AB018479) and Fel-2 (67%, AB018479). fTRGC4 including the intron and the CAN-SINE was also detected in the TraceArchive of feline genomic sequences ($\underline{\text{gnl}|\text{ti}|712587905}$) (NCBI, Bethesda, USA).

## 4. Discussion

We identified four additional TRGV and one additional TRGJ-region sequences within the TRACE Archive of feline genomic sequences. An additional TRGJ-region gene and a CAN-SINE were sequenced from feline cDNA using a SMART RACE procedure.

The four newly identified TRGV-region genes are probably pseudogenes due to mutations within their coding sequences and/or their RSS. One of the newly identified genes, fTRGV5P, represents a new subgroup of V-region genes within the feline TRGV repertoire according to the traditional threshold of 75% nucleotide homology [18] (see Table 1). It cannot be determined at the moment whether it lies within the feline TRG-locus or is an orphan gene.

The human TRG-locus contains 12 to 15 V-region genes, of which only four to six are functional V-region genes [10]. The remaining V-region genes are pseudogenes. Similarly our findings presented here add 4 V-region pseudogenes to the known 4 functional V-region genes in the cat.

Within the canine TRG-locus, there are 16 V-region genes and 8 of these are functional genes [19]. fTRGV5P is most similar to the canine subgroup TRGV6 (78% nucleotide homology), that has just one pseudogene member. Members of the feline subgroup TRGV4 are most similar to canine subgroup TRGV5 consisting of one pseudogene and one functional gene (For a comparison of feline and canine TRGV genes see the Supplementary Material available online at doi: /0.4061/ 2010/953272). A high degree of dissimilarity of V-region genes is almost characteristic of TRG-loci and can be also found in the dog and humans [15, 19].

In the newly characterized fTRGJ2.3 the nonamer of the 12RSS would be 5′- CATCTTTTT -3′, if the distance to the heptamer is 12 bp. This leaves a stretch of thymidine residues that is not flanked by one of the other three nucleotides at the 3′end. The putative nonamer of the RSS would be more similar to the consensus sequence if the spacer was only 10 bp long (see Figure 2(b), but this strongly impairs utilisation in V(D)J-recombination [17]. RSS can differ from the consensus sequence which is 5′-CACAGTG -3′ for the heptamer and 5′- ACAAAAACC -3′ for the nonamer (these consensus sequence is written as 3′RSS of V-region genes. 5′RSS of J-region genes are in reverse/complement) [5, 6]. Essential residues are the “ CAC ” at the beginning of the heptamer and the “G” at its end. In the nonamer, efficiency in recombination is enhanced by a cytosine-flanked adenine-rich core. Three adjacent A residues that are flanked by one of the other three nucleotides are essential for utilisation in V(D)J-recombination [17]. This is probably the reason why this sequence could only be found in the Trace Archive and not in our sequences, which are derived from transcribed genes.

When comparing fTRGJ2.4 to the other variants of the J-region subgroup fTRGJ2 it was apparent that it features a three bp long insertion near its 3′ end (see Figure 2(a). This insertion creates a potential 12RSS heptamer that is lacking a nonamer at the appropriate distance. The original 12RSS is positioned 12 bp upstream of the newly created heptamer. The molecular mechanism that gave rise to this construct remains unclear. As this clone is derived from a very young animal's thymus, it could be RNA of a developing T-cell prior to functional selection. 

Although some reports of insertional events within rearranged V-Domains exist in GenBank (accession numbers: M12859, M99577), no reports of J-region duplication and insertion or duplication and insertion in an inverted direction could be found. In the human T-cell line HPB-ALL an inversion of a segment containing a J-region in TRB is reported. This resulted in a J-D-J-construct that had its J-regions connected in head-to-head fashion [20]. However, this construct included two different J-regions. Moreover, translocations are often reported in antigen receptor loci and they may result in lymphomas because of activation of protooncogenes by translocated promoters and/or enhancers [5].

In addition to recombined TRG V-Domains, we also sequenced a pre-RNA containing a CAN-SINE. CAN-SINEs are derived from the tRNA for lysine and have an average length of 170 bp. They are made up of a tRNA-related part, a polypyrimidine region, and an A-rich tail. The latter two parts can differ in their length. In felids, CAN-SINEs typically contain two insertions [14]. The first can be found in all variants and lies upstream of the polypyrimidine region. The second can be found only in some of the variants and lies within the tRNA related part (not present in the CAN-SINE we sequenced). All other variants include a direct repeat downstream of the tRNA related part [14], as does the variant we describe.

Whether this SINE influences the functionality of fTRGC4 as a fTRGC-region gene is not known. It could only be found in unspliced form or in combination with fTRGJP. Additionally, it was twice detected in combination with fTRGJ3, the functionality of which is also questionable [9]. Another similar SINE was found 200 bp downstream of fTRGC1 exon 1 ($\underline{\text{gnl}|\text{ti}|749454182}$) with a homology of 77% to the CAN-SINE upstream of fTRGC4, of 80% to Fel-52, and of 75% to Fel-53.

## 5. Conclusions

In summary, we have found new V- and J-region sequences in the Trace Archives of feline genomic DNA. Though they are probably pseudogenes, they could partially be utilised in DNA conversion events and thereby become expressed genes. Because of this, their presence has to be kept in mind when designing or evaluating clonality studies of feline lymphomas or expression studies aimed at fTRG sequences. The unique sequence of the clone fTGII.2 might be a further clue to events that may occur during V(D)J recombination.

## Supplementary Material

The supplemental material provides a phylogenic dendrogram of canine and feline TRGV genes with human TRGV1 as out-group. From the canine genes only one member of each subgroup was included. Feline subgroup one (fTRGV1-3) is most closely related to canine subgroup 4 (cTRGV4-1) and 2 (cTRGV2-1). Canine subgroups 1, 3, 7 and 8 seem to have no known homologues within the feline repertoire. Feline subgroup 2 (fTRGV4) is most closely related to canine subgroup 5 (cTRGV5-1) and fTRGV5P is most closely related to canine subgroup 6 (cTRGV6-1). Maximum parsimony, bootstrap values are indicated next to the corresponding node.

## Figures and Tables

**Table 1:** Nucleotide homology of feline V-region genes.

	fTRG V2*	fTRG V3*	fTRG V4*	fTRG V4.1P**	fTRG V4.2P**	fTRG V4.3P**	fTRGV5P**
fTRGV1*	**79**%	**78**%	9%	8%	4%	18%	**68**%
fTRGV2*		**78**%	9%	6%	9%	22%	**71**%
fTRGV3*			40%	5%	12%	34%	**72**%
fTRGV4*				**88**%	**84**%	**86**%	6%
fTRGV4.1P**					82%	89%	5%
fTRGV4.2P**						84%	3%
fTRGV4.3P**							12%

* Previously published variants [8, 9]; ** newly identified variants.

**Table 2:** Nucleotide homology of feline J-region genes.

fTRGJ	2.2*	2.3**	2.4**	P^∗#^
2.1*	89%	79%	71%	76%
2.2*		86%	86%	83%
2.3**			95%	88%
2.4**				80%

* Previously published variants [8, 9]; ** newly identified variants. # TRGJ-region pseudogene.

## Abbreviations

V: Variable
D: Diversity
J: Joining
C: Constant
LINE: Long interspersed element
NHEJ: Nnonhomologous end joining
RAG: Recombination activating gene
RSS: Recombination signal sequence
SINE: Short interspersed element
TR: T-cell receptor
TRG: T-cell receptor *γ*
TRA: T-cell receptor *α*
fTRG: Feline T-cell receptor *γ*
RACE: Rapid amplification of cDNA ends
FR: Frame work region
CDR: Complementarity determining region.
