# Supplementary material for: Feline T-Cell Receptor γ V- and J-Region Sequences Retrieved from the Trace Archive and from Transcriptome Analysis of Cats
Source: Vet Med Int. 2010 Jun 16;2010:953272. doi: 10.4061/2010/953272 (PMC2904447; doi:10.4061/2010/953272)
Supplement: Supplementary file 1 — The supplemental material provides a phylogenic dendrogram of canine and feline TRGV genes with human TRGV1 as out-group. From the canine genes only one member of each subgroup was included. Feline subgroup one (fTRGV1-3) is most closely related to canine subgroup 4 (cTRGV4-1) and 2 (cTRGV2-1). Canine subgroups 1, 3, 7 and 8 seem to have no known homologues within the feline repertoire. Feline subgroup 2 (fTRGV4) is most closely related to canine subgroup 5 (cTRGV5-1) and fTRGV5P is most closely related to canine subgroup 6 (cTRGV6-1). Maximum parsimony, bootstrap values are indicated next to the corresponding node. [file 953272.f1.pdf]

Feline T-cell receptor γ V- and J-region sequences retrieved from the Trace Archive and from transcriptome analysis of cats – Supplemental Material

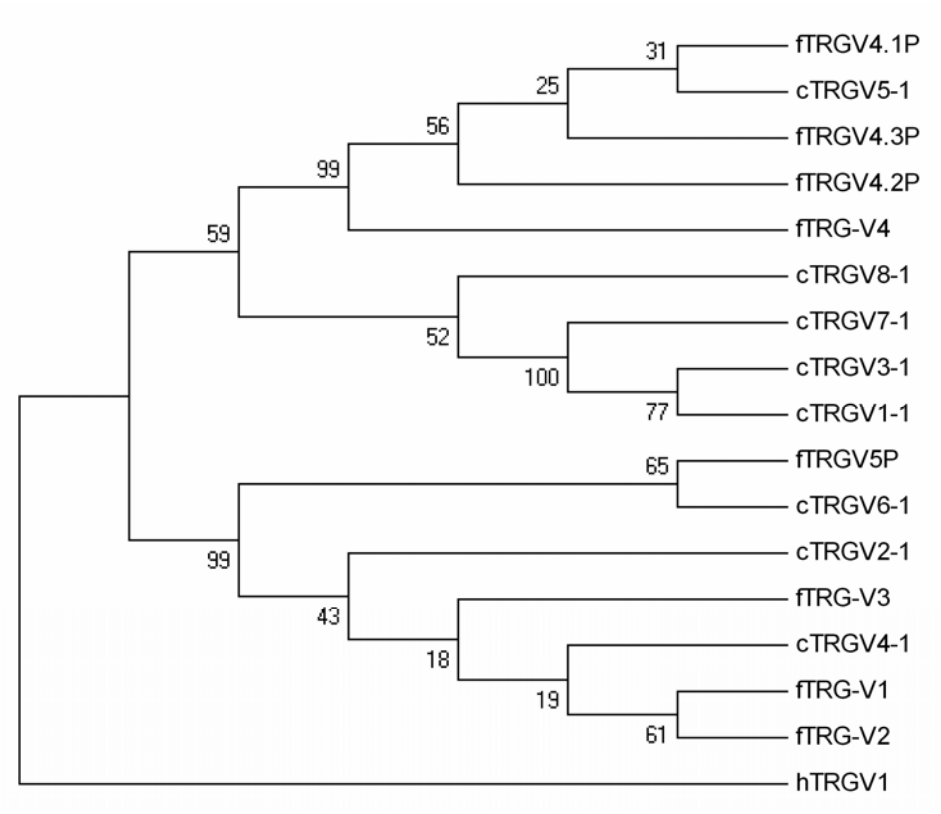

Supplemental Figure: Phylogenic dendrogram of canine and feline TRGV genes with human TRGV1 as out-group. From the canine genes only one member of each subgroup was included. Feline subgroup one (fTRGV1-3) is most closely related to canine subgroup 4 (cTRGV4-1) and 2 (cTRGV2-1). Canine subgroups 1, 3, 7 and 8 seem to have no known homologues within the feline repertoire. Feline subgroup 2 (fTRGV4) is most closely related to canine subgroup 5 (cTRGV5-1) and fTRGV5P is most closely related to canine subgroup 6 (cTRGV6-1). Maximum parsimony, bootstrap values are indicated next to the corresponding node.
